# Factors influencing plasma donation behavior of COVID‐19 recovered patients in Bangladesh: A pilot study

**DOI:** 10.1002/hsr2.974

**Published:** 2022-12-02

**Authors:** Nahid Salma, Md. Moyazzem Hossain, Sabina Yasmin, Muhammad Khairul Alam, Ahsan Rajvee Rimon, Jobaer Faruque, Mohammad Ali

**Affiliations:** ^1^ Department of Statistics Jahangirnagar University Savar Dhaka Bangladesh; ^2^ Directorate General of Health Services Mohakhali Dhaka Bangladesh

**Keywords:** antibodies sharing, convalescent plasma (cp), correlation matrix, factor analysis, Sars‐CoV‐2

## Abstract

**Background and Aim:**

The COVID‐19 pandemic has plagued our lives for more than 2 years, and the preference for convalescent plasma (CP) as a life‐saving treatment since CP has proven as a potential therapeutic option for acute COVID‐19 patients who were suffering from severe disease. It is important to identify which factors are associated with plasma donation. Therefore, this study aimed to assess the associated factors for CP donation to COVID‐19 patients.

**Methods:**

A cross‐sectional study was conducted online from December 21, 2021 to February 15, 2022 to identify different socio‐demographic factors and knowledge related to CP donation. People who recovered from the COVID‐19 infections and those who are willing to participate were included in the study. A total of 60 participants were included in the study. The data were analyzed using descriptive statistics, correlation matrix, and factor analysis.

**Results:**

The analysis results confirm that 41.67% (*n* = 25) of the participants aged 26–30 years; among the recovered patients, only about 23% (*n* = 14) of the participants donated plasma. Though 97% (*n* = 58) of the participants agreed to donate plasma when it will be needed, however, when someone asked to donate plasma then 76.67% (*n* = 46) of the patients declined it. Findings depict that gender had a weak positive relationship with ever decline in plasma donation at 5% level of significance and the age of the participants inversely related to plasma donation.

**Conclusion:**

Almost all the recovered participants were willing to donate plasma, however, due to a lack of knowledge and misconception, relatively few people actually did. This study reemphasizes the importance of health education to overcome the misconception about plasma donation, which is crucial for the treatment of COVID‐19 infection.

## 1 INTRODUCTION

The SARS‐CoV‐2 that is, COVID‐19, began to alarm the world in the first days of 2020 amid its initial flare‐up in China because of its severity.^1^ COVID‐19 was considered a new public health pandemic threatening the entire world with its rapid spread and causing deaths. On March 11, 2020, the World Health Organization (WHO) proclaimed that it was a worldwide pandemic due to the severity of the situation.^2^ The COVID‐19 pandemic impacted not only the health and well‐being but also other sectors of the country.^3^, ^4^, ^5^, ^6^, ^7^, ^8^, ^9^, ^10^ Comprehensive immunization is mandatory to avoid the widespread transmission of SARS‐CoV‐2 as well as they only can help prevent the poor outcome of the disease. We are fortunate enough that the vaccine was developed, however, at an early stage, different alternative ways were used to stop it's spreading and combat it. A previous study highlighted that different types of actions were taken to reduce cases and fatalities all over the world.^11^ A previous study in China revealed critical upgrades in patients influenced by the Coronavirus and treated with convalescent plasma (CP).^12^ In the earlier period of the spread of these COVID‐19 viruses, numerous endeavors have been made to treat wiped‐out patients utilizing off‐label drugs. A year after the start of the COVID‐19 pandemic, invented vaccination met people's high expectations and created immunizations that shield from SARS‐CoV‐2, the infection that causes COVID‐19. However, vaccination is one of the most successful approaches to ward against and manage infectious diseases. Now it becomes a big challenge to make these vaccines available to cover all the people worldwide.^13^, ^14^

One of the world's first promising treatments for COVID‐19 patients is the use of CP because it may help to recover the patients who were admitted to hospitals.^15^ The CP could be used as a supplement to antiviral treatment.^16^ So CP may be deployed within weeks of the commencement of future epidemics or pandemics caused by infectious diseases.^16^, ^17^ Moreover, Dr. Jed Gorlin, adjunct professor in the Department of Laboratory Medicine and Pathology at Hennepin Healthcare and Children's Hospitals and Clinics of Minnesota, said that “The risk of giving one unit of plasma is minimal, and as far as specifics, we're learning after the fact.”^18^ CP therapy was widely practiced by clinicians in Bangladesh during the first and second waves of the pandemic, so we wanted the investigate the factors influencing the plasma donation behavior among the recovered patients at that time.

The government of Bangladesh has established the “Shohojoddha” plasma network to simplify the availability and accessibility of plasma from coronavirus patients. Under this effort, many public and private sector partners collaborated to develop the platform.^19^ Doctors at numerous hospitals in Bangladesh were prescribing plasma therapy to their ICU patients who are moderately or seriously infected.^20^ However, no in‐depth studies were found in the literature judging the attitudes toward plasma donation in the context of Bangladesh. Moreover, it is necessary to know people's attitudes toward plasma donation. Plasma donation is influenced by several factors, like motivations, beliefs, and attitudes. Furthermore, plasma donation decisions are enticed by covariates such as gender, age, marital status, occupation, residence, levels of education, wealth index, social behavior, and the desire to save human life. Therefore, the authors were targeted to assess the perceptions and motivations of CP donors. The authors conducted this study to determine people's awareness and perceptions toward CP donation and to identify factors that may motivate them.

## 2 METHODOLOGY

**Figure 1:**
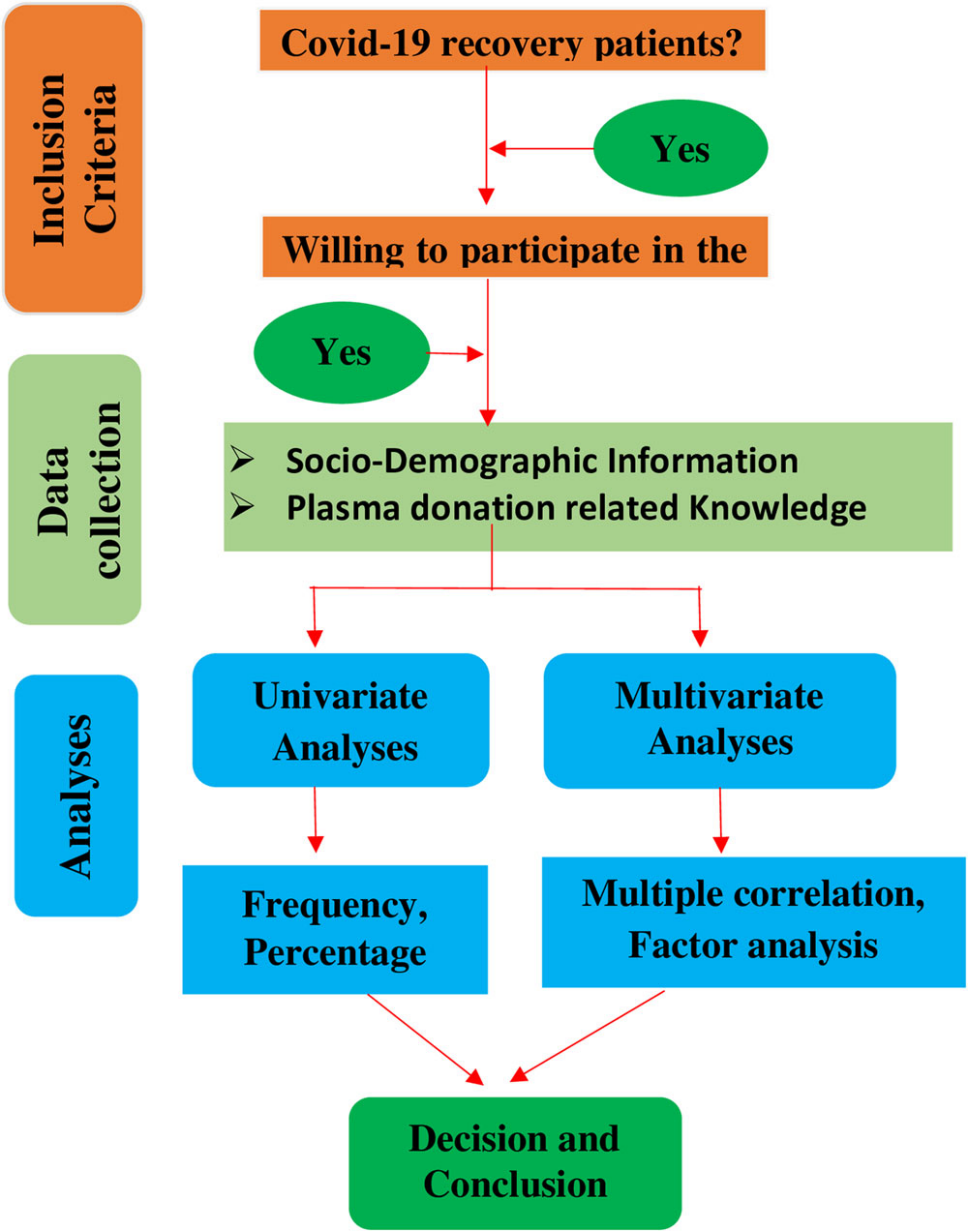
Flowchart of the study

The study was conducted among COVID‐19 recovery patients in Bangladesh between December 21, 2021 and February 15, 2022 through an online survey. Before involvement in this survey, the authors informed the respondents about the objective of this study using phone calls, Facebook Messenger, and WhatsApp, and made sure that the information they provided would be reserved privately and no identifiable information will not be disclosed along with oral consent was taken. Participants who were willing to participate and contribute to this study were then sent a link to the designed Google form along with instructions to complete it. The link to the designed Google form was shared with participants via email and various social media groups. The respondents finally agreed to provide authorization for their data to be used in the study. The whole study design is described by a flowchart presented in Figure 1.

There are several guiding principles for determining the right sample size for pilot research so that different features of interest, such as effect size, the standard deviation of the outcome measure, reliability, or confidence level, may be determined with adequate precision.^21^, ^22^, ^23^, ^24^ However, Viechtbauer et al.^25^ described a straightforward method to estimate the required sample size for a pilot study, with a preferred level of confidence and a specified probability.^25^ Researchers used the following formula for pilot study.^26^ Bearing in mind, Considering a 95% confidence level (γ=0.95) with probability (π=0.05), the essential sample size for this pilot survey has been determined by the following formula,

$$n=\frac{\ln\left(1-\gamma \right)}{\ln\left(1-\pi \right)}.$$

Hence, this formula suggests that a sample of size 59 is sufficient for a pilot survey. A total of 60 participants were included in the final analysis after discarding the five incomplete responses.

Several demographic characteristics of the participants were included in this study, like the age of the respondents, marital status (married, unmarried, divorced), gender (male, female), educational level (higher secondary, graduated and above), wealth index (poor, middle class, rich), occupation (student, housewife, service, business, and others), place of residence (urban, rural), living with (alone, family members, family, and children). Moreover, plasma donation‐related data was structured so that respondents felt free to answer easily. This section consists of information such as: the blood group of the respondent, ever donated plasma, willingness to donate plasma, declination of donating plasma, reasons behind omitting plasma donation, motivational factors behind plasma donation, and the necessity of a token gift for the donor.

To investigate the data, a set of statistical tools has been applied. Firstly, descriptive data analysis consisting of frequencies and percentages is performed for socio‐demographic information and plasma donation‐related knowledge. A range of univariate and multivariate statistical techniques have been applied to determine the desired results. In multivariate analysis, we used factor analysis with an orthogonal factor model to identify latent factors that help for finding the amount of variance among observed variables. Varimax with the Kaiser Normalization method was used as a rotation method for the simplicity of the factor matrix. Bartlett's test of sphericity is statistically significant with (*p* < 0.01), and the Kaiser‐Meyer‐Olkin measure of sampling adequacy is 0.536, providing evidence of the appropriateness of factor analysis in this pilot study. the statistical package for social science version‐26, Stata version‐15, and Microsoft Excel were used to analyze the data.

## 3 RESULTS

A total of 60 participants were included in the study; about two‐fifth (42%) of the participants were in the age range of 26–30 years, and two‐thirds (60%) of them were married. The majority (81.67%) of the respondents had an education level of graduate or above, and the same proportion of the respondents belonged to middle‐class wealth status. Among 60 participants, half of the respondents were doing services, and one‐third of them were students. A major portion of the respondents resided in urban areas (83.33%), and 45% lived with their families and children in their houses. Only 3.33% of the respondents have an AB‐blood group. Out of 60 participants, only 14 participants have donated plasma though the majority of the respondents (97%) agreed to donate plasma when it will be needed, however, when someone asked to donate plasma then about 76.67% of the respondents declined to donate plasma due to some reason and the main reasons behind the decline in donating plasma were fear (54.35%), health problems (30.43%), family restrictions (10.87%), and religious reasons (4.35%). Three in four people think a gift token should be given to plasma donors to encourage them (Table 1).

**Table 1:** Characteristics of different factors associated with plasma donation

Variable	Categories	Frequency (*n*)	Percent (%)
Age (years)	21–25	12	20.00
	26–30	25	41.67
	31–35	11	18.33
	36–40	7	11.67
	More than 40	5	8.33
Marital status	Married	36	60.00
	Unmarried	24	40.00
Gender	Male	39	65.00
	Female	21	35.00
Education status	Higher secondary	11	18.33
	Graduate and above	49	81.67
Wealth Index	Middle class	49	81.67
	Rich	11	18.33
Occupation	Student	19	31.67
	Housewife	4	6.67
	Service	30	50.00
	Business	4	6.67
	Others	3	5.00
Residence	Urban	50	83.33
	Rural	10	16.67
Living with	Family	56	93.33
	Alone	4	6.67
Blood group	B+	22	36.67
	O+	20	33.33
	AB+	16	26.67
	AB–	2	3.33
Will you donate plasma if needed?	Yes	58	96.67
	No	2	3.33
Donate plasma	Yes	14	23.33
	No	46	76.67
Have you ever decline to donate plasma when asked by someone?	Yes	46	76.67
	No	14	23.33
If declined then, reasons of decline	Fear	25	54.35
	Religious reason	2	4.35
	Health problem	14	30.43
	Family restrictions	5	10.87
A token gift should be offered to plasma doners	Yes	45	75.00
	No	15	25.00

**Figure 2:**
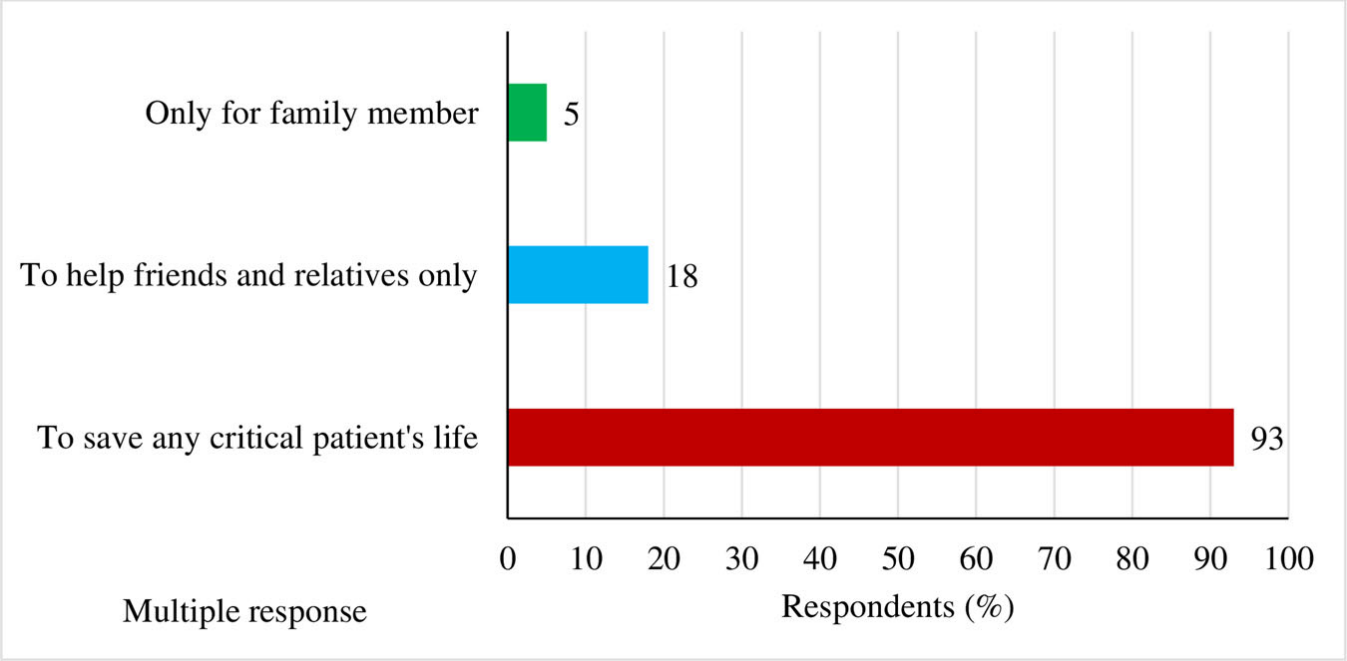
Motivation factor behind plasma donation (multiple responses)

The findings depict that most respondents (93%) thought that the motivational factor behind plasma donation is to save a critical patient's life, while 18% of the participants thought it was to help only friends and relatives and considered it as a social responsibility. A few (5%) of respondents prioritized their families only but were not willing to donate plasma to others. Here, the author's accepted multiple responses (Figure 2).

The correlation matrix was demonstrated to identify the association among all the following variables: age ($x_{1}$), marital status ($x_{2}$), gender ($x_{3}$), educational status ($x_{4}$), wealth index ($x_{5}$), occupation ($x_{6}$), residence ($x_{7}$), living with ($x_{8}$), blood group ($x_{9}$), donate plasma ($x_{10}$), will donate plasma if needed ($x_{11}$), preference for plasma donation ($x_{12}$), decline to donate plasma ($x_{13}$), reasons of decline ($x_{14}$), a token gift should give ($x_{15}$). The association asserted that some variables had a significant linear relationship with others. Findings depict that gender had a weak positive relationship with ever decline in plasma donation at 5% level of significance and the age of the participants inversely related to plasma donation. Moreover, plasma donation is positively associated with a token gift offered to the donors though it is insignificant at 5% level of significance (Table 2). The correlation matrix leads to doing factor analysis to find the linear relationship and the amount of variance among observed variables.

**Table 2:** Matrix of association of the selected variables

	x_1_	x_2_	x_3_	x_4_	x_5_	x_6_	x_7_	x_8_	x_9_	x_10_	x_11_	x_12_	x_13_	x_14_	x_15_
x_1_	1														
x_2_	–0.584**	1													
x_3_	–0.172	0.042	1												
x_4_	0.188	–0.140	0.103	1											
x_5_	–0.078	0.052	0.103	0.002	1										
x_6_	0.670**	–0.640**	–0.235	0.294*	–0.213	1									
x_7_	0.088	0.091	0–.234	–0.250	0.019	0.088	1								
x_8_	0.315*	–0.221	–0.128	0.239	–0.029	0.239	–0.060	1							
x_9_	0.022	0.064	–0.024	–0.003	–0.078	–0.073	–0.205	0.182	1						
x_10_	–0.049	0.128	0.239	0.044	0.159	–0.070	–0.070	–0.182	–0.068	1					
x_11_	0.104	–0.031	0.152	0.108	–0.108	0.036	–0.102	0.031	–0.018	0.126	1				
x_12_	0.163	0.037	0.058	0.088	–0.088	0.106	–0.083	–0.025	–0.102	0.102	0.809**	1			
x_13_	0.040	–0.216	–0.296*	–0.152	–0.213	0.325	0.221	–0.019	–0.246	–0.222	–0.162	–0.065	1		
x_14_	–0.078	0.163	0.353**	0.082	0.109	–0.253	–0.139	0.054	0.264*	0.073	0.343**	0.140	0.818**	1	
x_15_	–0.098	0.001	0.141	0.074	–0.074	–0.054	–0.154	0.015	0.246	0.227	0.220	0.107	–0.081	0.180	1

* *p* < 0.05.

** *p* < 0.01.

**Figure 3:**
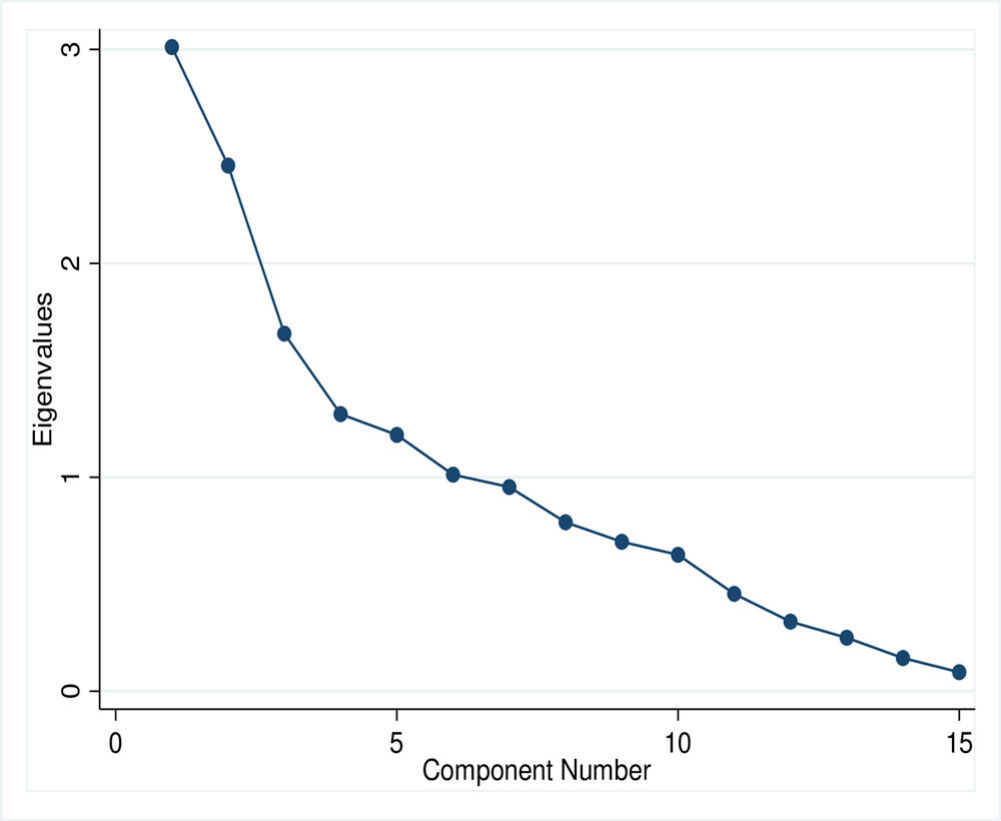
Scree plot of eigenvalues after factor analysis

The scree plot of eigenvalues is used to select the number of factors based on the size of the eigenvalues, which is visualized in Figure 3. This scree plot shows that the first five factors account for most of the total variability in the data. The first five components have eigenvalues greater than 1. We may consider these strong factors. After that, eigenvalues of component 6 and onwards were dropdown slowly, that is, an elbow has been observed in component 5, that is, the excess factors represent an exceptionally small extent of the changeability and are likely insignificant.

After Varimax rotation had been performed on the data, from the following rotated factor matrix, we could assert that Factor 1 has high positive factor loadings for occupation (0.820), age (0.818), educational status, who living with and high negative loadings for marital status (–0.794), so this factor describes the patient's demographic characteristics. Factor 2 differentiates reasons for declining to donate plasma and ever declining to donate plasma with high factor loadings, so this factor may be labeled as the reasons behind declining. Factor 3 has high positive factor loadings for preference for plasma donation and will donate plasma if needed, so this factor might be depicted as the perspective of plasma donation. Factor 4 included variables like a token that should be given to donors, blood groups, and place of residence, called basic information. Lastly, factor 5 has high factor loadings for donating plasma, gender, and wealth index, which may represent plasma donation (Table 3).

**Table 3:** Results of Factor analysis

Variables	Factor 1	Factor 2	Factor 3	Factor 4	Factor 5
Occupation	0.820				
Age	0.818				
Marital status	–0.794				
Educational status	0.493				
Whom living with	0.467				
Ever decline to donate plasma		0.900			
Reasons for declining to donate plasma		–0.862			
Preference for plasma donation			0.924		
Will you donate plasma if needed			0.915		
A token gift should be offered to the plasma donator				0.720	
Place of residence				–0.620	
Blood group				0.555	
Donate plasma					0.724
Gender					0.541
Wealth index					0.426
Eigenvalue	2.488	2.196	1.916	1.539	1.495
Explained variance	16.588	14.637	12.774	10.258	9.966

*Note*: Extraction method: principal component analysis.

Rotation method: Varimax with Kaiser Normalization.

## 4 DISCUSSION

The quick start‐up of a COVID‐19 CP program worked well^27^; however, the COVID‐19 pandemic had a negative impact on blood donation.^28^ This article provides an opportunity to explore the main motivations and barriers to blood donation and highlight some key concerns relating to CP donation. Many factors influence the willingness to donate plasma. However, this study articulates and summarizes people's awareness and preference for plasma donation. The result of this pilot study demonstrated that 97% of the COVID‐19 recovery patients showed a positive preference for plasma donation, whereas about 23% of the patients donated CP. According to the patients, their ultimate motivation behind plasma donation is to save one's life (93%). Besides, 76.67% of the respondents declined to donate plasma for crucial reasons. Researchers pointed out that respondents aged 18–30 years, male and having blood group “O” Rh D positive gave a maximum contribution to plasma donation.^29^ They noted that the “Fear Factor of Contracting the Disease” has been a significant obstacle in inspiring and persuading COVID‐recovered patients to donate plasma.

The results from correlation analysis indicated that some variables had a significant relationship with others. The findings of the factor analysis depicted that some variables contribute positively and some negatively to the variation of the study variable. Generic donation anxieties were inversely connected to the intention to donate plasma, although a deeper sense of compassion via adversity and moral and civic duty were positively related.^30^ Fear of health, family pressure, and a new procedure were the top three reasons for the refusal of plasma donation.^31^ Donating plasma during the COVID‐19 pandemic was linked with perceived risk, severity, anxiety, and response cost.^32^ Moreover, a lack of empathy is a key factor in discouraging blood donation.^28^ The public's willingness could be increased by raising awareness and disseminating information about the importance of COVID‐19 CP. Moreover, the government and policymakers should give importance to health education to all people to overcome the misconception about plasma donation.

CP therapy treatment has been utilized since the mid‐1900s to treat irresistible infections, in the absence of antivirals and monoclonal antibodies, the CP can be treated as a life‐saving treatment.^33^ CP is being studied worldwide, as a treatment for COVID‐19 and prevention, however, it did not reduce all‐cause mortality.^34^, ^35^, ^36^ Researchers recommend that CP treatment could be a viable alternative in the event of a COVID‐19 emergency.^37^ However, there is no evidence of benefit shown by several trials with COVID‐19 patients, therefore, it is rarely practiced nowadays.

## 5 STRENGTH AND LIMITATIONS

The strength of this study is that it is the first study in Bangladesh however, it has some limitations. Firstly, since it is a pilot study, the sample size is very small, which may not represent the overall country's situation. Secondly, face‐to‐face data collection was not possible considering the pandemic situation as a result the study may have some sample selection bias.

## 6 CONCLUSION

Although Bangladesh started using CP for severe and critical COVID‐19 patients, there are still myths, misconceptions, and a lack of knowledge about plasma donation, which has prevented people from becoming CP donors. Although the majority of patients who have recovered are willing to donate plasma, more than a fifth of respondents have only done so. Despite myths and misconceptions, different motivational campaigns should be launched to demonstrate that plasma donation is safe and we may save someone's life by donating plasma.

## AUTHOR CONTRIBUTIONS

**Nahid Salma**: Conceptualization; methodology; supervision; writing – original draft; writing – review and editing. **Md Moyazzem Hossain**: Conceptualization; methodology; supervision; visualization; writing – review and editing. **Sabina Yasmin**: Conceptualization; data curation; formal analysis; visualization; writing – original draft. **Muhammad Khairul Alam**: Conceptualization; data curation; formal analysis; visualization; writing – original draft. **Ahsan Rajvee Rimon**: Conceptualization; data curation; writing – original draft. **Jobaer Faruque**: Conceptualization; data curation; writing – original draft. All authors have read and approved the final version of the manuscript.

## CONFLICT OF INTEREST

The authors declare no conflict of interest. Furthermore, none of the authors participating in this research communication has any connections with other people or organizations that could impact (bias) the findings in an unethical way.

## TRANSPARENCY STATEMENT

The lead author Md. Moyazzem Hossain affirms that this manuscript is an honest, accurate, and transparent account of the study being reported; that no important aspects of the study have been omitted; and that any discrepancies from the study as planned (and, if relevant, registered) have been explained.

## ETHICS STATEMENT

The authors inform participants about the study's goal and assure them that the information they provide will be kept confidential, and they obtain oral agreement before participating in the survey. The participants were told that their names and other identifying information would remain anonymous. This study was conducted entirely online in accordance with the requirements of the Helsinki Declaration of the human participant research association.

## Data Availability

The authors confirm that the data supporting the findings of this study are available within the article, and data will be available upon a reasonable request to the corresponding author.

## References

[1] Zhou G , Zhao Q . Perspectives on therapeutic neutralizing antibodies against the novel coronavirus sars‐cov‐2. Int J Biol Sci. 2020;16(10):1718‐1723. 32226289 10.7150/ijbs.45123 PMC7098029

[2] Cucinotta D , Vanelli M . WHO declares COVID‐19 a pandemic. Acta Biomed. 2020;91(1):157‐160. 32191675 10.23750/abm.v91i1.9397 PMC7569573

[3] Thunström L , Newbold SC , Finnoff D , Ashworth M , Shogren JF . The benefits and costs of using social distancing to flatten the curve for COVID‐19. J Benefit‐Cost Anal. 2020;11(2):179‐195.

[4] Ahmed H.U . Economic ramifications of Covid‐19 in Bangladesh. Global Times [Internet] . May 11, 2020. Accessed May 25, 2021. https://www.globaltimes.cn/page/202005/1187977.shtml

[5] Yeasmin S , Banik R , Hossain S , et al. Impact of COVID‐19 pandemic on the mental health of children in Bangladesh: a cross‐sectional study. Child Youth Serv Rev. 2020;117:105277. 32834275 10.1016/j.childyouth.2020.105277 PMC7387938

[6] Yasmin S , Alam MK , Ali FB , Banik R , Salma N . Psychological impact of COVID‐19 among people from the banking sector in Bangladesh: a cross‐sectional study. Int J Ment Health Addict. 2021;20(3):1485‐1499. 33495689 10.1007/s11469-020-00456-0 PMC7816746

[7] Alam MK , Ali FB , Banik R , Yasmin S , Salma N . Assessing the mental health condition of home‐confined university level students of Bangladesh due to the COVID‐19 pandemic. J Public Heal. 2021;30:1685‐1692. 10.1007/s10389-021-01542-w PMC8053029 33898164

[8] Muller AE , Hafstad EV , Himmels JPW , et al. The mental health impact of the covid‐19 pandemic on healthcare workers, and interventions to help them: a rapid systematic review. Psychiatry Res. 2020;293:113441. 32898840 10.1016/j.psychres.2020.113441 PMC7462563

[9] Rahman, A. , Abdulla, F. , Karimuzzaman, M. , & Hossain, M. M . (2022). Burden of COVID‐19 on health and wellbeing, education, and economy of Bangladesh. Clinical Case Reports, 10(11), e06639. Portico. 10.1002/ccr3.6639 PMC9684679 36439388

[10] Hossain, Md. M. , Abdulla, F. , Karimuzzaman, Md. , & Rahman, A. (2020). Routine Vaccination Disruption in Low‐Income Countries: An Impact of COVID‐19 Pandemic. Asia Pacific Journal of Public Health, 32(8), 509–510. 10.1177/1010539520957808 32917115

[11] Abdulla F , Nain Z , Karimuzzaman M , Hossain MM , Rahman A . A non‐linear biostatistical graphical modeling of preventive actions and healthcare factors in controlling COVID‐19 pandemic. Int J Environ Res Public Health. 2021;18(9):4491. 33922634 10.3390/ijerph18094491 PMC8122857

[12] Cai X , Ren M , Chen F , Li L , Lei H , Wang X . Blood transfusion during the COVID‐19 outbreak. Blood Transfus. 2020;18:79‐82. 32267830 10.2450/2020.0076-20 PMC7141939

[13] Musa S , Dergaa I , Abdulmalik MA , Ammar A , Chamari K , Saad HB . BNT162b2 COVID‐19 vaccine hesitancy among parents of 4023 young adolescents (12–15 years) in Qatar. Vaccines. 2021;9(9):981. 34579218 10.3390/vaccines9090981 PMC8473301

[14] Moyazzem Hossain, Md. , Abdulla, F. , & Rahman, A. (2022). Challenges and difficulties faced in low‐ and middle‐income countries during COVID‐19. Health Policy OPEN, 3, 100082. 10.1016/j.hpopen.2022.100082 36405972 PMC9642028

[15] FDA. Donate COVID‐19 plasma [Internet]. 2020. Accessed December 20, 2021. https://www.fda.gov/emergency-preparedness-and-response/coronavirus-disease-2019-covid-19/donate-covid-19-plasma

[16] Yiğenoğlu TN , Hacıbekiroğlu T , Berber İ , et al. Convalescent plasma therapy in patients with COVID‐19. J Clin Apheresis. 2020;35(4):367‐373. 32643200 10.1002/jca.21806 PMC7361338

[17] Klassen SA , Senefeld JW , Senese KA , et al. Convalescent plasma therapy for COVID‐19: a graphical mosaic of the worldwide evidence. Front Med. 2021;8:684151. https://pubmed.ncbi.nlm.nih.gov/34164419/ 10.3389/fmed.2021.684151 PMC8215127 34164419

[18] University of Minnesota Medical Students , Nagarajan E , Kanwar S . U blood‐banking experts explain how convalescent plasma can be used to treat COVID‐19 [Internet]. News and events, University of Minnesota Medical School. Medical School—University of Minnesota. 2020. Accessed July 25, 2022. https://med.umn.edu/news-events/u-blood-banking-experts-explain-how-convalescent-plasma-can-be-used-treat-covid-19

[19] Antara NF . Govt launches plasma donation platform to connect Covid‐19 patients. Dhaka. Tribune [Internet]. June 9, 2020. Accessed May 24, 2021. https://archive.dhakatribune.com/health/coronavirus/2020/06/09/govt-launches-plasma-donation-platform-to-connect-covid-19-patients

[20] Haque MA , Amin R . Expediting convalescent plasma availability in Bangladesh. The Daily Star [Internet]. April 13, 2021. Accessed May 24, 2021. https://www.thedailystar.net/opinion/news/expediting-convalescent-plasma-availability-bangladesh-2076653

[21] Hertzog MA . Considerations in determining sample size for pilot studies. Res Nurs Health. 2008;31(2):180‐191. 18183564 10.1002/nur.20247

[22] Johanson GA , Brooks GP . Initial scale development: sample size for pilot studies. Educ Psychol Meas. 2010;70(3):394‐400.

[23] Julious SA . Sample size of 12 per group rule of thumb for a pilot study. Pharm Stat. 2005;4(4):287‐291.

[24] Thabane L , Ma J , Chu R , et al. A tutorial on pilot studies: the what, why and how. BMC Med Res Methodol. 2010;10:1. 20053272 10.1186/1471-2288-10-1 PMC2824145

[25] Viechtbauer W , Smits L , Kotz D , et al. A simple formula for the calculation of sample size in pilot studies. J Clin Epidemiol. 2015;68(11):1375‐1379. 26146089 10.1016/j.jclinepi.2015.04.014

[26] Salma N , Hossain MM , Yasmin S , Alam MK , Alam KMR . Assessing mental health status among COVID‐19 recovery patients in Bangladesh—a pilot study. Int J Ecol Econ Stat. 2022;43(3):46‐60.

[27] Fabricius MM , Dandachi D . COVID‐19 convalescent plasma: from donation to treatment—a systematic review & single center experience. Mo Med. 2021;118(1):74‐80 33551490 PMC7861598

[28] Gkirtsou C , Konstantinidis T , Cassimos D , et al. Views and attitudes of blood donors toward blood donation during the COVID‐19 pandemic in thrace region, Greece. Int J Environ Res Public Health. 2022;19(9):4963. https://pubmed.ncbi.nlm.nih.gov/35564360/ 35564360 10.3390/ijerph19094963 PMC9101185

[29] Mahapatra S , Pati S . Constraints and challenges in convalescent plasma collection amidst the Covid 19 pandemic—strategies and recommendations to overcome these. Transfus Clin Biol. 2021;28(2):175‐179. 33677086 10.1016/j.tracli.2021.02.003 PMC7931723

[30] Masser BM , Ferguson E , Thorpe R , et al. Motivators of and barriers to becoming a COVID‐19 convalescent plasma donor: a survey study. Transfus Med. 2021;31(3):176‐185. 33368777 10.1111/tme.12753

[31] Dhiman Y , Coshic P , Pandey HC , et al. Deterrents in recruitment of COVID‐19 convalescent plasma donors: experience from a hospital‐based blood centre in India. Transfus Med. 2021;31(3):149‐154. https://pubmed.ncbi.nlm.nih.gov/33749020/ 33749020 10.1111/tme.12768 PMC8251325

[32] Masser BM , Hyde MK , Ferguson E . Exploring predictors of Australian community members' blood donation intentions and blood donation‐related behavior during the COVID‐19 pandemic. Transfusion. 2020;60(12):2907‐2917. 32905630 10.1111/trf.16067

[33] Focosi D , Anderson AO , Tang JW , Tuccori M . Convalescent plasma therapy for covid‐19: state of the art. Clin Microbiol Rev. 2020;33(4):e00072‐20. 32792417 10.1128/CMR.00072-20 PMC7430293

[34] Varma A , Dergaa I , Mohammed AR , et al. Covid‐19 and diabetes in primary care—how do hematological parameters present in this cohort? Expert Rev Endocrinol Metab. 2021;16(3):147‐153. 33818239 10.1080/17446651.2021.1909472

[35] Dergaa I , Abubaker M , Souissi A , et al. Age and clinical signs as predictors of COVID‐19 symptoms and cycle threshold value. Libyan J Med. 2022;17(1):2010337. 34895104 10.1080/19932820.2021.2010337 PMC8667934

[36] Axfors C , Janiaud P , Schmitt AM , et al. Association between convalescent plasma treatment and mortality in COVID‐19: a collaborative systematic review and meta‐analysis of randomized clinical trials. BMC Infect Dis. 2021;21(1):1170. 34800996 10.1186/s12879-021-06829-7 PMC8605464

[37] Zhao Q , He Y . Challenges of convalescent plasma therapy on COVID‐19. J Clin Virol 2020;127:104358. 32305026 10.1016/j.jcv.2020.104358 PMC7146649

